# Supplementary material for: Validation of the T-Lymphocyte Subset Index (TLSI) as a Score to Predict Mortality in Unvaccinated Hospitalized COVID-19 Patients
Source: Biomedicines. 2022 Nov 2;10(11):2788. doi: 10.3390/biomedicines10112788 (PMC9687349; doi:10.3390/biomedicines10112788)
Supplement: Supplementary file 1 [file biomedicines-10-02788-s001.zip › biomedicines-1939009-supplementary.pdf]

Table S1: Multivariable logistic regression analysis for the composite endpoint represented by ICU-admission/in-hospital 30-day mortality in patients with SARS-CoV-2 infection

Parameter	Odds Ratio	OR 95% Confidence Interval		p
		Lower bound	Upper bound	
Sex (M)	2.441	1.005	5.931	**0.049**
Age	1.006	0.976	1.038	0.685
N# of comorbidities	1.381	1.051	1.815	**0.020**
Delta Symp-TBNK	0.881	0.809	0.959	**0.003**
LDH	1.007	1.004	1.011	**< 0.001**
IL-6	1.006	0.997	1.014	0.185
TLSI	2.114	1.283	3.486	**0.003**

The analysis was performed considering the composite event of either ICU admission or death within 30 days from hospital admission because of COVID-19. Statistically significant p values are highlighted in bold. ICU: intensive care unit; OR: odds ratio; M: male; N#: number; Delta Symp-TBNK: days between symptoms' onset and peripheral blood T-, B-, NK-cell assessment; LDH: lactate dehydrogenase; IL-6: interleukin-6; TLSI: T-Lymphocyte subset index

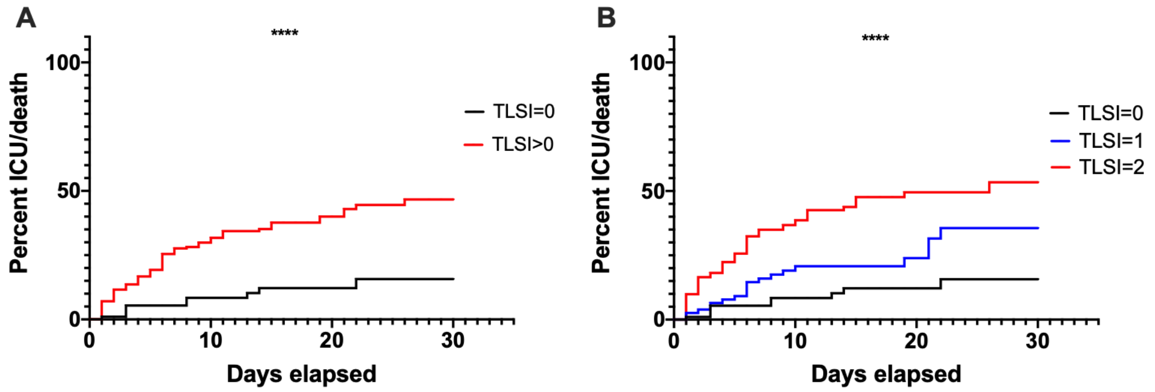

Figure S1: Time-to-event analysis considering the composite event ICU-admission/30-day in-hospital mortality in COVID-19 patients stratified according to TLSI score

Time-to-event analysis considering the composite event ICU-admission/30-day in-hospital mortality in COVID-19 patients, stratified into two (A) and three (B) groups, according to TLSI score, ranging from 0 to 2.
The analysis was performed with the Log-rank (Mantel-Cox) test. COVID-19 patients with a TLSI>0 had an increased risk of either ICU-admission or 30-day in hospital mortality, with a hazard ratio of 2.75 compared to COVID-19 patients with TLSI=0 (Log-rank Mantel-Cox test p<0,0001) (A).
The log-rank test for trend was also used for the time-to-event analysis of the cohort after stratification into three groups, showing a statistically significant trend from TLSI=0 to TLSI=2, p<0.0001 (B).
ICU: intensive care unit; TLSI: T-Lymphocyte subset index; ****: p<0.0001
